# Peripheral Giant Cell Granuloma Associated With an Impacted Tooth and Prosthetic Irritation: A Multifactorial Etiological Insight and Surgical Management Challenge

**DOI:** 10.7759/cureus.108636

**Published:** 2026-05-11

**Authors:** Mohammed Farouk, Oussama Bentahar

**Affiliations:** 1 Odontology Department, Faculty of Medicine, Pharmacy, and Dentistry, Sidi Mohamed Ben Abdellah University, Fes, MAR

**Keywords:** impacted canine, inflammation, mandible, oral and maxillofacial surgery, peripheral giant cell granuloma

## Abstract

Peripheral giant cell granuloma (PGCG) is a reactive oral lesion typically associated with local irritants; however, the interactions among multiple concurrent etiological contributors remain poorly characterized in atypical presentations. We report a 55-year-old female patient presenting with a large exophytic mass in the posterior mandible. Clinical examination revealed a reddish-purple sessile lesion in contact with edentulous areas and a removable prosthesis, suggesting chronic mechanical irritation. Radiographic assessment demonstrated localized bone resorption and a vertically impacted mandibular canine adjacent to the lesion, raising suspicion of a more aggressive pathology. The initial clinical diagnosis was epulis. Surgical management included complete excision of the lesion, extraction of compromised teeth, removal of the impacted tooth, and curettage of the underlying bone. Histopathological analysis confirmed PGCG, characterized by a vascular fibrocellular stroma with numerous multinucleated giant cells. No recurrence was observed after a two-year follow-up, with satisfactory mucosal healing and functional outcome. This case suggests a possible synergistic interaction between prosthetic irritation, previous extraction, and a vertically impacted mandibular canine in promoting lesion growth and atypical features, including bone involvement. Comprehensive management that addresses both the lesion and its etiological factors appears essential for optimizing outcomes and reducing the risk of recurrence.

## Introduction

Peripheral giant cell granuloma (PGCG) is a reactive, nonneoplastic lesion arising from the gingiva or alveolar mucosa, commonly attributed to chronic local irritation [1,2]. It is generally associated with single etiological factors such as dental plaque, calculus, poorly adapted restorations, or trauma following tooth extraction [3,4]. Clinically, PGCG presents as a nodular exophytic mass with variable coloration, while radiographic findings are often limited or nonspecific. Despite its benign nature, larger lesions or those associated with underlying bone involvement may mimic more aggressive pathologies, complicating diagnosis and management [1,5].

While the role of individual irritative factors has been widely reported, the potential synergistic effect of multiple concurrent etiological contributors remains insufficiently explored [2,6]. In particular, the combined influence of prosthetic irritation, impacted teeth, and postextraction changes on lesion development and progression is not well established in the literature.

While PGCG is commonly associated with local irritative factors, most reported cases emphasize a single etiological contributor. The potential synergistic interactions among multiple concurrent factors, such as prosthetic irritation, impacted teeth, and postextraction changes, remain insufficiently explored. Understanding these combined effects is essential, as they may influence lesion size, behavior, and diagnostic complexity. In this context, we report an atypical case of PGCG associated with multiple local factors, with the aim of highlighting a multifactorial etiological pathway and its clinical implications.

## Case presentation

A 55-year-old female patient was referred to the oral surgery department for evaluation of a progressively enlarging mass in the posterior mandibular region. The lesion had appeared approximately one year after the extraction of a mandibular tooth and had shown gradual, continuous growth. The patient denied pain, spontaneous bleeding, or functional discomfort. Her medical history was unremarkable, and no systemic conditions or medication use were reported.

**Figure 1:**
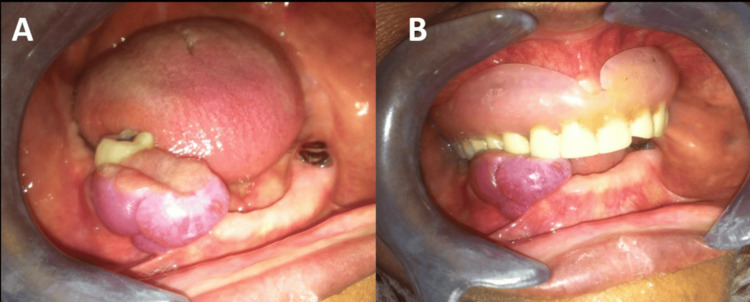
Clinical findings (A) Frontal intraoral view showing a large exophytic reddish-purple lesion occupying the posterior edentulous mandibular ridge adjacent to a compromised mandibular molar. The lesion exhibits a lobulated surface and broad sessile implantation. (B) Lateral intraoral view demonstrating the spatial relationship between the lesion and the removable maxillary prosthesis. Visible indentation marks on the lesion surface suggest chronic prosthetic irritation and repetitive mechanical trauma

Intraoral examination revealed a well-circumscribed, exophytic lesion measuring approximately 4 cm in its greatest dimension, located in the posterior mandible and extending across edentulous areas corresponding to the previously extracted teeth. The lesion exhibited a reddish to brownish coloration with a smooth surface and a sessile base. On palpation, it was soft to firm in consistency and nontender. Notably, the lesion showed direct contact with a removable maxillary prosthesis, with visible impressions on its surface, suggesting chronic mechanical irritation. The adjacent dentition included a compromised mandibular molar and residual root fragments (Figures 1A, 1B).

**Figure 2:**
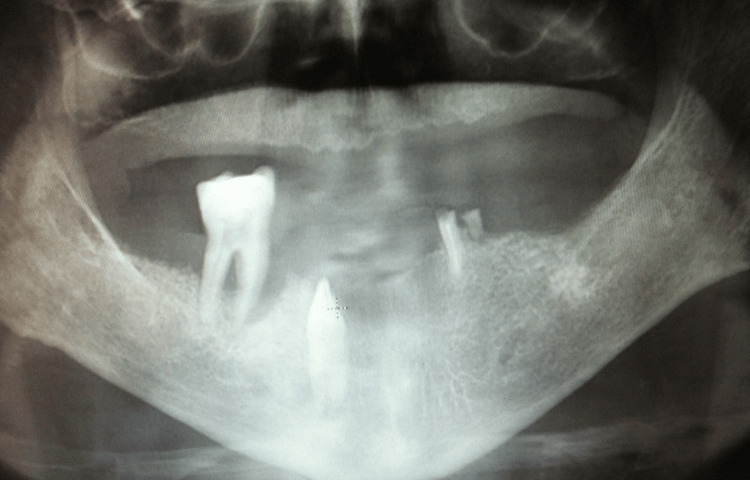
Radiographic findings Panoramic radiograph demonstrating localized bone resorption in the posterior mandibular region, associated with residual roots and presence of a vertically impacted canine adjacent to the lesion

Panoramic radiographic examination demonstrated localized horizontal bone resorption underlying the lesion, along with terminal bone loss affecting the adjacent molar. In addition, a vertically impacted mandibular canine was identified in close proximity to the lesion, with its crown positioned adjacent to the base of the lesion, suggesting a potential local irritative contribution. No clearly defined intraosseous lesion was observed, supporting a peripheral origin with secondary bone involvement (Figure 2). Based on the clinical and radiographic findings, a provisional diagnosis of epulis was established, with differential diagnoses including pyogenic granuloma, peripheral ossifying fibroma, and central giant cell granuloma with cortical perforation.

Considering the lesion size, progressive growth pattern, associated bone involvement, and the coexistence of multiple local irritative factors, a comprehensive surgical approach was planned. Under local anesthesia, a full-thickness mucoperiosteal flap was elevated to provide adequate visualization and access to both the lesion and the underlying bone structures. Complete excision of the lesion was performed in toto with careful preservation of the surrounding tissues.

In addition to lesion removal, the compromised mandibular molar and residual root fragments were extracted because they represented potential chronic inflammatory and irritative sources that could contribute to lesion persistence or recurrence. The vertically impacted mandibular canine, located in close proximity to the lesion base, was also surgically removed. Although PGCG is considered a peripheral reactive lesion, the impacted tooth was considered a possible contributing factor due to its intimate anatomical relationship with the lesion and its potential role in maintaining chronic local inflammation.

**Figure 3:**
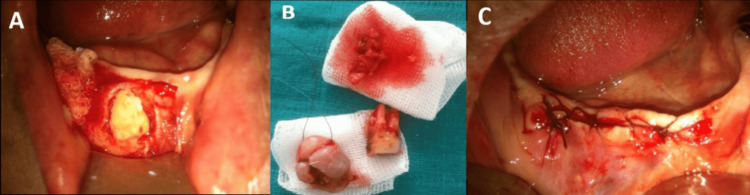
Surgical management (A) Intraoperative view following mucoperiosteal flap elevation, showing exposure of the lesion site and underlying bone with evidence of bone involvement. (B) Excised surgical specimen, including the soft lesion and extracted teeth, illustrating the nodular morphology and size of the lesion. (C) Immediate postoperative view after complete excision of the lesion and sutures

Thorough curettage of the underlying bone was subsequently performed to eliminate any residual reactive tissue and reduce the risk of recurrence, particularly given the radiographic evidence of secondary bone involvement. The surgical site was then irrigated with saline solution and closed with interrupted sutures to promote optimal healing and tissue adaptation (Figures 3A-3C).

**Figure 4:**
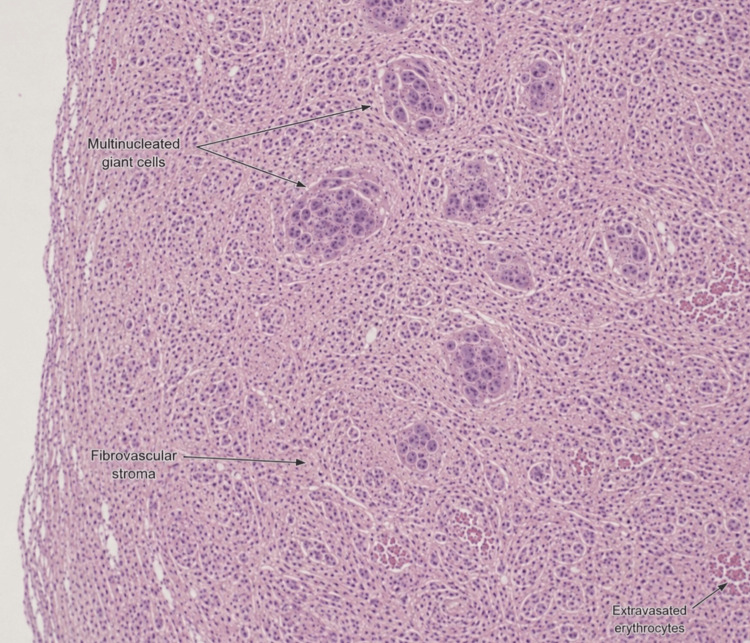
Histopathological findings Histopathological examination of the lesion. Low-power photomicrograph showing a fibrovascular stroma containing numerous multinucleated giant cells dispersed within spindle-shaped mesenchymal cells and focal extravasated erythrocytes, consistent with peripheral giant cell granuloma (H&E stain, ×40) H&E: hematoxylin and eosin

Histopathological examination at low magnification revealed a fibrovascular stroma containing numerous multinucleated giant cells distributed within a background of spindle-shaped mesenchymal cells and focal extravasated erythrocytes, consistent with PGCG (Figure 4). No signs of recurrence were detected over a two-year follow-up period, with satisfactory mucosal healing and functional outcome.

## Discussion

PGCG is widely regarded as a benign reactive lesion arising from the gingiva or alveolar mucosa, typically in response to local irritative stimuli [3,7]. While most cases are associated with a single contributing factor, the present report highlights a more complex scenario in which multiple local elements appear to act synergistically, influencing both lesion development and clinical behavior. This multifactorial context is particularly relevant in explaining the unusually large size of the lesion, its progressive growth, and the associated bone changes observed radiographically.

In the current case, three principal local factors were identified: a previous tooth extraction site, chronic mechanical irritation from a removable prosthesis, and the presence of an impacted tooth in close proximity to the lesion. Each of these factors has been individually implicated in the pathogenesis of PGCG. However, their coexistence in a single anatomical site suggests a cumulative or synergistic effect rather than independent contributions. The lesion developed in an edentulous mandibular region subjected to repeated mechanical trauma, as evidenced by visible prosthetic imprints on its surface. This supports the role of persistent mucosal irritation as a key driver of reactive tissue proliferation.

From a pathophysiological perspective, although a direct causal relationship cannot be definitively established based on a single case, it is plausible that the coexistence of multiple local irritative factors contributed to a sustained inflammatory microenvironment. Chronic mechanical irritation from the prosthesis, combined with inflammatory stimuli from the extraction site and the impacted tooth, may enhance local cytokine release and osteoclastic activity. This process could promote the recruitment and proliferation of multinucleated giant cells within a vascularized fibrocellular stroma, which is the characteristic of PGCG.

The presence of a vertically impacted mandibular canine adjacent to the lesion adds complexity. Although PGCG is not an intraosseous lesion, impacted teeth may contribute to localized inflammation, alter bone architecture, and serve as a persistent source of irritation [8,9]. This association may also complicate diagnostic interpretation, particularly when radiographic findings reveal bone involvement. In such situations, distinguishing PGCG from more aggressive entities, such as central giant cell granuloma or other osteolytic lesions, becomes critical. In the present case, the absence of a well-defined intraosseous lesion, combined with histopathological confirmation, supported the diagnosis of a peripheral lesion with secondary bone involvement.

Therapeutically, this case underscores the importance of a comprehensive surgical approach. While simple excision may be sufficient for small, localized lesions [10], the presence of multiple etiological factors necessitates a more extensive intervention. In this patient, complete excision of the lesion was combined with removal of all potential sources of irritation, including extraction of compromised teeth, removal of the impacted tooth, and curettage of the lesion base. This approach is consistent with the reactive nature of PGCG and aims to minimize the risk of recurrence by addressing both the lesion and its underlying causes.

The therapeutic strategy adopted in this case was intentionally more extensive than simple lesion excision because the lesion exhibited several atypical features, including large size, progressive enlargement, and radiographic bone involvement. In addition, the coexistence of multiple potential etiological factors raised concern regarding the persistence of chronic local inflammatory stimulation. Consequently, management was directed not only toward removal of the reactive lesion itself but also toward elimination of all possible contributing irritative sources.

The extraction of the impacted canine was considered particularly relevant because impacted teeth may alter local tissue architecture, promote chronic inflammation, and maintain continuous mechanical or inflammatory stimulation in adjacent soft tissues. Similarly, curettage of the underlying bone was performed to eliminate residual reactive tissue and minimize the risk of recurrence. This comprehensive approach may explain the absence of recurrence during the two-year follow-up period observed in the present case. The absence of recurrence after two years of follow-up supports the effectiveness of this strategy.

From a clinical standpoint, this case highlights the need for a systematic and multidisciplinary evaluation of PGCG, particularly in atypical or extensive presentations. Clinicians should actively investigate the presence of multiple local irritative factors, including prosthetic trauma, residual roots, impacted teeth, and postextraction changes. Failure to identify and eliminate these contributing factors may lead to incomplete treatment and an increased risk of recurrence. Furthermore, prosthetic reassessment following surgery is essential to prevent continued mucosal irritation.

This case provides three important clinical take-away messages. First, PGCG may present with atypical features, including significant size enlargement and secondary bone involvement, when multiple local irritative factors coexist. Second, impacted teeth located adjacent to reactive gingival lesions may contribute to chronic inflammation and should be carefully evaluated during diagnostic assessment. Third, successful management of extensive PGCG requires not only lesion excision but also the elimination of all associated etiological factors to minimize the risk of recurrence.

The main limitation of this report is its single-case nature, which precludes definitive conclusions regarding causality. Nevertheless, it provides valuable insight into the potential role of multifactorial etiological interactions in PGCG and emphasizes the importance of comprehensive management to achieve favorable clinical outcomes.

## Conclusions

PGCG should be regarded as a reactive lesion whose clinical presentation may be significantly influenced by the coexistence of multiple local etiological factors. This case illustrates how the combined effects of prosthetic irritation, previous tooth extraction, and the presence of an impacted tooth can contribute to lesion enlargement, atypical features, and diagnostic complexity. Accurate diagnosis relies on integrating clinical, radiographic, and histopathological findings, particularly in cases with bone involvement that may mimic more aggressive entities. From a therapeutic perspective, complete surgical excision alone may be insufficient if underlying contributing factors are not addressed. Comprehensive management, including the elimination of all potential sources of chronic irritation, appears essential for minimizing recurrence risk and ensuring favorable outcomes. This case underscores the importance of a systematic and multifactorial approach to the evaluation and management of PGCG, especially in atypical or extensive presentations.
